# Image mosaicking using SURF features of line segments

**DOI:** 10.1371/journal.pone.0173627

**Published:** 2017-03-15

**Authors:** Zhanlong Yang, Dinggang Shen, Pew-Thian Yap

**Affiliations:** 1 School of Marine Science and Technology, Northwestern Polytechnical University, Xi’an, Shaanxi, China; 2 Department of Radiology and Biomedical Research Imaging Center (BRIC), University of North Carolina, Chapel Hill, NC, United States of America; 3 Department of Brain and Cognitive Engineering, Korea University, Seoul, Korea; Banner Alzheimer’s Institute, UNITED STATES

## Abstract

In this paper, we present a novel image mosaicking method that is based on Speeded-Up Robust Features (SURF) of line segments, aiming to achieve robustness to incident scaling, rotation, change in illumination, and significant affine distortion between images in a panoramic series. Our method involves 1) using a SURF detection operator to locate feature points; 2) rough matching using SURF features of directed line segments constructed via the feature points; and 3) eliminating incorrectly matched pairs using RANSAC (RANdom SAmple Consensus). Experimental results confirm that our method results in high-quality panoramic mosaics that are superior to state-of-the-art methods.

## 1 Introduction

The automatic construction of large, high-resolution image mosaics is an active area of research in the fields of photogrammetry, computer vision, image processing, and computer graphics [1]. It is considered as important as other image processing tasks such as image fusion [2], image denoising [3], image segmentation [4] and depth estimation [5]. Image mosaicking finds applications in a wide variety of areas. A typical application is the construction of large aerial and satellite images from collections of smaller photographs [1, 6]. More applications include scene stabilization and change detection [7], video compression [8], video indexing [9] and so on [1]. Some widely used commercial software packages for image mosaicking are available, such as AutoStitch [10], Microsoft ICE [11], and Panorama Maker [12].

The key problem in image mosaicking is to combine two or more images by stitching them seamlessly together into a new one that distorts the original images as little as possible [13]. Image mosaicking techniques can be mainly divided into two categories: grayscale-based methods and feature-based methods. Grayscale-based methods are easy to implement, but they are relatively sensitive to grayscale changes especially under variable lighting. Feature-based methods extract features from image pixel values. Because these features are partially invariant to lighting changes, matching ambiguity can be better resolved during image matching. Matching robustness can be further improved by using feature points that can be detected reliably. Many methods have been shown to be effective for the extraction of image feature points, for example, Harris method [14], Susan method [15], and Shi-Tomasi method [16]. Feature-based image mosaicking methods afford two main advantages: (1) the computation complexity of image matching will be significantly reduced since the number of feature points is far smaller than the number of pixels; (2) the feature points are very robust to unbalanced lighting and noise, resulting in better image mosaicking results.

**Fig 1:**
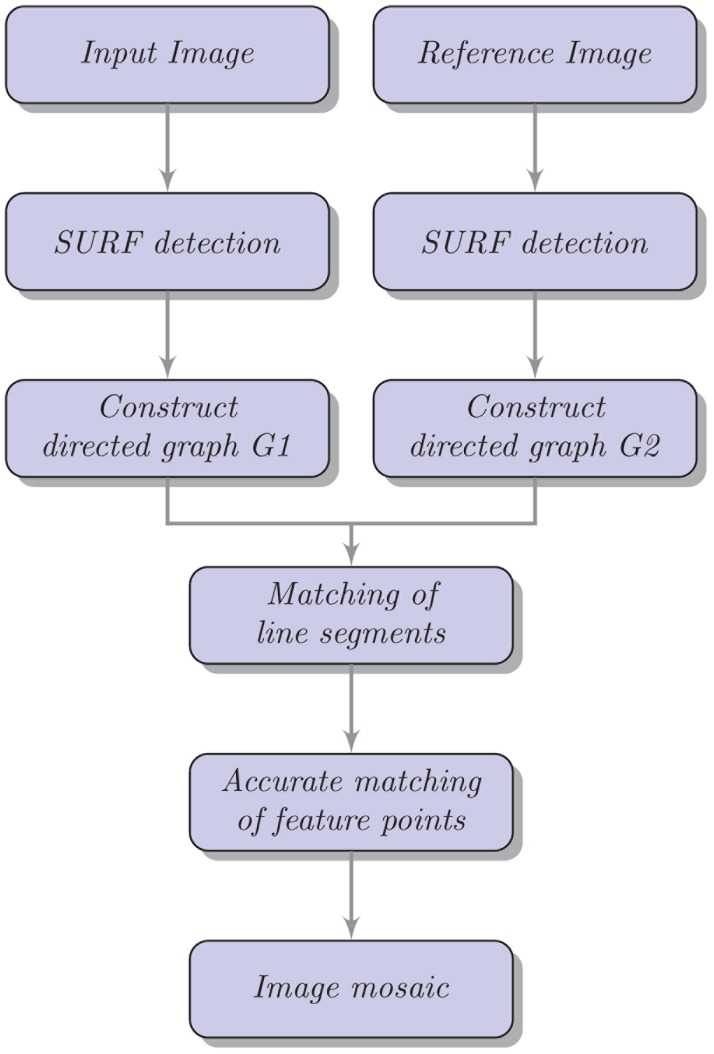
An overview of our method.

A wide variety of feature detectors and descriptors have been proposed in the literature (e.g. [17–21]). Detailed comparisons and evaluations of these detectors and descriptors on benchmark datasets were performed in [22, 23]. Among various methods, SIFT [18] has been shown to give the best performance [22]. Recent efforts (e.g. SURF [24], BRISK [25], FREAK [26], NESTED [27], and Ozuysal’s method [28]) have been focused on improving SIFT-based matching accuracy and reducing computation time. Arguably SURF [24] is among the best methods. Fei Lei et al. proposed a fast method for image mosaicking based on a simple application of SURF [29]. Jun Zhu et al. proposed an image mosaicking method that uses the Harris detector and SIFT features of line segments [30]. For performance and efficiency, this method uses Harris corner detection operator to detect key points. Then features of line segments are used to match feature points owing to their effective representation of local image information, such as textures and gradients. However, the Harris corner detector is very sensitive to changes in image scale; so it does not provide a good basis for matching images of different sizes. Motivated by this observation, we propose an image mosaicking method that is based on SURF features [24] of line segments. First, the method uses the SURF detection operator to locate feature points and then constructs a directed graph of the extracted points. Second, it describes directed line segments with SURF features and matches them to obtain rough matching of points. Finally, it adjusts matching points and eliminates incorrectly matched pairs through the RANSAC algorithm [31]. The framework of our method is summarized in Fig 1.

## 2 SURF

SURF, like the SIFT operator, is a robust feature detection method that is invariant to image scaling, rotation, illumination changes, and even substantial affine distortion. Both of these descriptors encode the distribution of pixel intensities in the neighborhoods of the detected points. SURF is computationally more efficient than SIFT owing to the use of integral images [32] and the box filters [33] that approximate second order partial derivatives of Gaussian convolutions. Similarly to many other approaches, SURF consists of two consecutive parts, including feature point detection and feature point description.

### 2.1 SURF feature-point detector

Similarly to the SIFT method, the detection of features in SURF relies on a scale-space representation combined with first and second order differential operators. The key feature of the SURF method is that these operations are approximated using box filters computed via integral images. So, the procedure of SURF feature detection involves first computing an integral image, establishing an image scale space with box filters, and finally locating feature points in the scale space.

The SIFT detector is based on the determinant of the Hessian matrix, which is defined at point **x** = (*x*, *y*) and scale *σ* as

$$\begin{array}{c} H\left(x,\sigma \right)=\left[\begin{array}{cc} L_{xx}\left(x,\sigma \right) & L_{xy}\left(x,\sigma \right)\\ L_{xy}\left(x,\sigma \right) & L_{yy}\left(x,\sigma \right) \end{array}\right], \end{array}$$ (1)

where *L*_*xx*_(**x**, *σ*) is the convolution of the Gaussian second order derivative $\frac{\partial^{2}}{\partial x^{2}}g\left(\sigma \right)$ with the image *I* at point **x**, and similarly for *L*_*xy*_(**x**, *σ*) and *L*_*yy*_(**x**, *σ*). As mentioned before, in order to reduce computation, SURF approximates *L*_*xx*_, *L*_*xy*_, *L*_*yy*_ with the box filtering using sum of the Haar wavelet responses, resulting respectively in *D*_*xx*_, *D*_*xy*_, *D*_*yy*_ and

$$\begin{array}{c} H_{\text{approx}}\left(x,\sigma \right)=\left[\begin{array}{cc} D_{xx}\left(x,\sigma \right) & D_{xy}\left(x,\sigma \right)\\ D_{xy}\left(x,\sigma \right) & D_{yy}\left(x,\sigma \right) \end{array}\right]. \end{array}$$ (2)

This can be performed very efficiently using an integral image *I*_∑_, which given an input image *I* is calculated as

$$\begin{array}{c} I_{\sum }\left(x\right)=\sum_{i=0}^{i\leq x}\sum_{j=0}^{j\leq y}I\left(i,j\right). \end{array}$$ (3)

The determinant of the approximated Gaussians is

$$\begin{array}{c} \text{det}\left(H_{\text{approx}}\right)=D_{xx}D_{yy}-{\left(0.9D_{xy}\right)}^{2}. \end{array}$$ (4)

Thus, the interest points, including their scales and locations, are detected in approximate Gaussian scale space. The size of the box filter is varied with octaves and intervals [34]:

$$\begin{array}{c} \text{Filter}\;\text{Size}=3\times \left(2^{\text{octave}}\times \text{interval}+1\right). \end{array}$$ (5)

**Fig 2:**
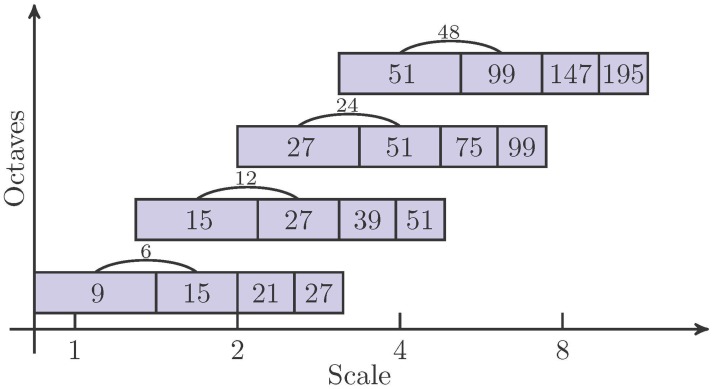
Filter sizes for four different octaves and intervals (marked by arcs).

The filter sizes for various octaves and intervals are illustrated in Fig 2. Only pixels with greater responses than their surrounding pixels are classified as interest points. The maximal responses are then interpolated in scale and space to locate interest points with sub-pixel accuracy.

### 2.2 SURF descriptor

The goal of a descriptor is to provide a unique and robust description of the intensity distribution within the neighborhood of the point of interest. In order to achieve rotational invariance, the orientation of the point of interest needs to be determined. Orientation is calculated in a circular area of radius 6s centered at the interest point, where s is the scale at which the interest point is detected. In this area, Haar wavelet responses in *x* and *y* directions are calculated and weighted with a Gaussian centered at the point of interest. By computing the sum of the horizontal and vertical responses within a sliding orientation window of size π/3 and traversing the entire circle every 5 degrees, 72 orientations can be obtained. The two summed responses then yield a local orientation vector. The longest of such vector over all windows defines the main orientation.

Once position, scale and orientation are determined, a feature descriptor is computed. The first step consists of constructing a square region centered around the feature point and oriented along the orientation determined previously. The region is divided uniformly into smaller 4 × 4 sub-regions. For each sub-region, Haar wavelet responses are computed at 5 × 5 regularly-spaced sample points. The *x* and *y* wavelet responses, denoted by *dx* and *dy* respectively, are computed at these sample points weighting with a Gaussian centered at the interest point and summed up over each sub-region to form a first set of entries to the feature vector. In order to obtain information on the polarity of the intensity changes, the sums of the absolute values of the responses, |*dx*| and |*dy*|, are also extracted. Therefore each sub-region is associated with a four-dimensional vector

$$\begin{array}{c} v=\left(\sum dx,\sum dy,\sum \left|dx\right|,\sum \left|dy\right|\right). \end{array}$$ (6)

Combining the vectors, v’s, from all sub-region yields a single 64-dimensional descriptor, which is normalized to unit-norm for contrast invariance.

## 3 Matching of directed line segments

### 3.1 Rough matching

The best candidate match for each keypoint is found by identifying its nearest neighbor in the set of keypoints generated from a reference image. The nearest neighbor is defined as the keypoint with the minimal Euclidean distance determined based on the invariant descriptor vector described above.

**Fig 3:**
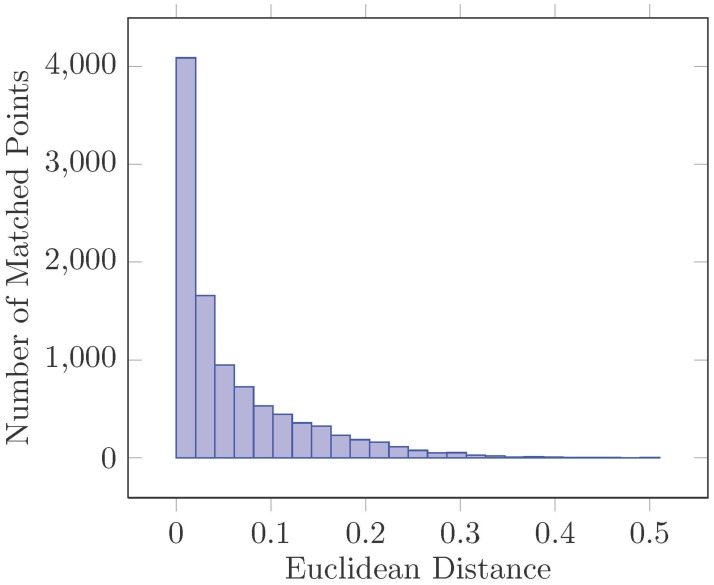
Euclidean distances of 10000 matched keypoints.

However, many features from an image do not have any matching counterparts in the reference image because they arise from background clutter or cannot be detected in the reference image. Therefore, we use a global threshold on the distance to discard keypoints without good matches. Fig 3 shows the Euclidean distance of 10000 keypoints with correct matches for real image data. This figure was generated by matching images with different scales, rotation angles, changes in illumination, and affine distortions. As shown in Fig 3, most of the matched pairs have small Euclidean distances ranging from 0 to 0.15. We set the global threshold to 0.1 in our experiments, eliminating more than 90% of the false matches while discarding less than 5% of the correct matches.

### 3.2 Line segment features

Features of line segments are effective representation of local image information, such as textures and gradients. Given two images *I* and *I*′ to be matched, the feature points are detected for each image using SURF to construct two directed graphs, *G* = (*V*, *E*) and *G*′ = (*V*′, *E*′), where *V* = {*a*_1_, *a*_2_,⋯,*a*_*n*_} and *V*′ = {*b*_1_, *b*_2_,⋯,*b*_*m*_} are key points extracted from *I* and *I*′, and *E* = {(*a*_*i*_, *a*_*j*_), *i* ≠ *j*} and *E*′ = {(*b*_*i*_, *b*_*j*_), *i* ≠ *j*} are the edge sets of directed graphs *G* and *G*′, respectively. Features are generated for each line segment between two key points. For each edge of graph *G*, *e*_*ij*_ ∈ *E*, with starting point *a*_*i*_ and end point *a*_*j*_, we equidistantly sample three points {*p*_1_, *p*_2_, *p*_3_}, with *p_k_* = *p_i_* + ((*k*−1)/2) (*p_j_*−*p_i_*), *k* = 1, 2, 3. *p*_*i*_ is the coordinates of point *a*_*i*_. The SURF features are extracted for each of these points, giving a feature matrix *S* = [*s*_1_, *s*_2_, *s*_3_]. Each *s*_*k*_ is a 64-dimensional vector. For each line segment, we have a 192-dimensional feature vector.

### 3.3 Nearest neighbor matching

We use the nearest-neighbor matching criterion proposed in [30] for rough matching of line segments. Assuming image *I* has *n*_1_ directed line segments, *L* = [*l*_1_, *l*_2_,⋯,*l*_*n*_1__], and image *I*′ has *n*_2_ directed line segments, $L^{\prime }=[l_{1}^{\prime },l_{2}^{\prime },\ldots ,l_{n_{2}}^{\prime }]$, the nearest-neighbor pairs can be encoded using an adjacency matrix $K\in R^{n_{1}\times n_{2}}$:

$$\begin{array}{c} K\left(i,j\right)=\left\{\begin{array}{cc} 1 & l_{j}^{\prime }\;\text{is}\;\text{the}\;\text{nearest}\;\text{neighbor}\;\text{of}\;l_{i}\\ 0 & \text{otherwise}. \end{array}\right. \end{array}$$ (7)

The distance between a pair of line segments *l*_*i*_ and $l_{j}^{\prime }$, with feature matrices *S*_*i*_ and $S_{j}^{\prime }$ respectively, is defined using the *F*-norm of the feature matrices: $d\left(l_{i},l_{j}^{\prime }\right)={\left\|S_{i}-S_{j}^{\prime }\right\|}_{F}$.

The matching is further refined as follows. With the sets of key points in two given images, *V* = {*a*_1_, *a*_2_,⋯,*a*_*n*_} and *V*′ = {*b*_1_, *b*_2_,⋯,*b*_*m*_}, we use the statistical voting method reported in [30] to obtain the matching frequency of each point. A matrix *G* ∈ *R*^*n*×*m*^ is initiated as a null matrix. If based on *K* two straight lines match each other, we vote for the starting point pairs and the ending point pairs of the two lines once. This is carried out by incrementing the corresponding element in *G* by 1. A larger element in matrix *G* indicates higher probability of matching of two points. The procedure for computation of matrix *G* is detailed in Algorithm 1.

**Algorithm 1** Computation of *G*

Input: Matrix *K*

Output: Matrix *G*

1: **procedure** ComputeMatrix(*K*, *G*)

2:  Initialize *G* ∈ *R*^*m*×*n*^ as a null matrix

3:  **for** *i* = 1, 2,…,*n*_1_, *j* = 1, 2,…,*n*_2_ **do**

4:   **if** *K*(*i*, *j*) = 1 **then**

5:    Find directed line segment *l*_*i*_[*a*_*l*_ → *a*_*m*_], $l_{j}^{\prime }[b_{p}\to b_{q}]$

6:    *G*(*l*, *p*) = *G*(*l*, *p*) + 1, *G*(*m*, *q*) = *G*(*m*, *q*) + 1

7:   **end if**

8:  **end for**

9:  Output matrix *G*

10: **end procedure**

To avoid matching to too many points to one point, the criteria to select matching points are as follows:

- Discard pairs with *G*(*i*, *j*) ≤ *σ*, where *σ* = 0.5 max_*i*, *j*_ *G*(*i*, *j*).
- Select pairs giving maximal values in all rows and columns as matched pairs.
- If the maximal element in row *i* and the maximal element in column *j* are not the same, select the larger one. For example, assuming *G*(*i*, *p*) is the maximal element in row *i* and *G*(*q*, *j*) is the maximal element in column *j*, if *G*(*i*, *p*) > *G*(*q*, *j*), then *a*_*i*_ and *b*_*p*_ match each other.

Incorrectly matched pairs are further removed by using RANSAC (RANdom SAmple Consensus) [31] and then a homography matrix **M** is estimated for image alignment.

## 4 Experimental results

In this section, the experimental results of the proposed method are presented. Evaluation was performed with gray level images with different rotation angles, scales, illumination, and affine distortions are used. Representative results are shown here.

**Fig 4:**
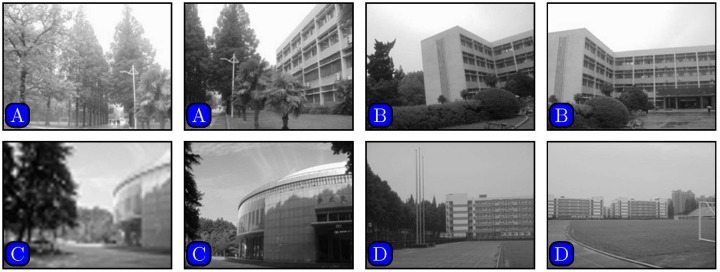
Image pairs with photometric or geometric variations. (A) lighting, (B) rotation, (C) blur, (D) scaling. Reprinted from [30] under a CC BY license, with permission from [Computational and Mathematical Methods in Medicine], original copyright [2014].

In order to compare our proposed method with a recent state-of-the-art method presented in [30], images downloaded from the website [35] were used. Representative image pairs are shown in Fig 4. The lighting conditions in the two images are largely different in Fig 4(A). The left image has longer exposure time than the right one. The two images in Fig 4(B) were taken by ordinary camera in different orientations. The two images have different resolutions in Fig 4(C). The left one is a blurred low-resolution image and the right one has higher resolution. In Fig 4(D), the left image is taken with the lens of the camera zoomed relative to the right one. Therefore, the buildings in the left image appear larger than the ones in the right.

**Fig 5:**
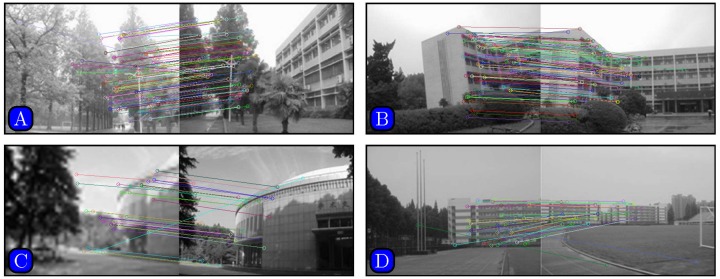
Matching results based on SURF method.

**Fig 6:**
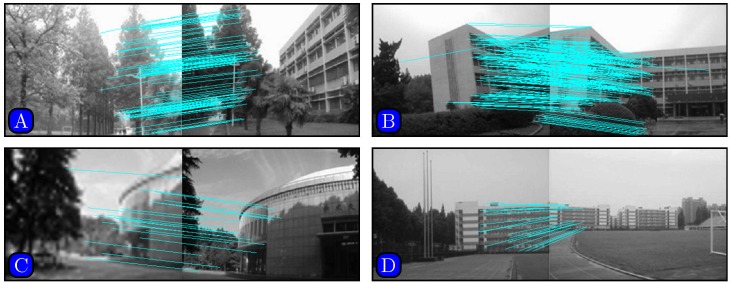
Matching results based on SIFT method.

**Fig 7:**
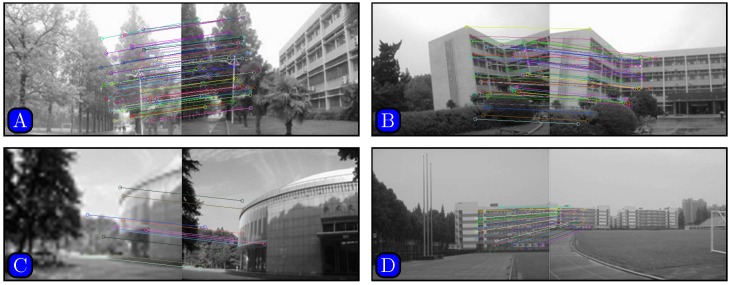
Matching results based on proposed method.

Results of matching by different methods are shown in Figs 5–7. Fig 5 indicates that SURF cannot even stitch the images correctly due to incorrectly matched points. Figs 6 and 7 demonstrate that both SIFT and our method obtain good results. However, Fig 6(B) indicates that SIFT still results in wrongly matched points. Our method incorporates robust statistical voting and rough matching strategies that could eliminate incorrectly matched pairs.

**Fig 8:**
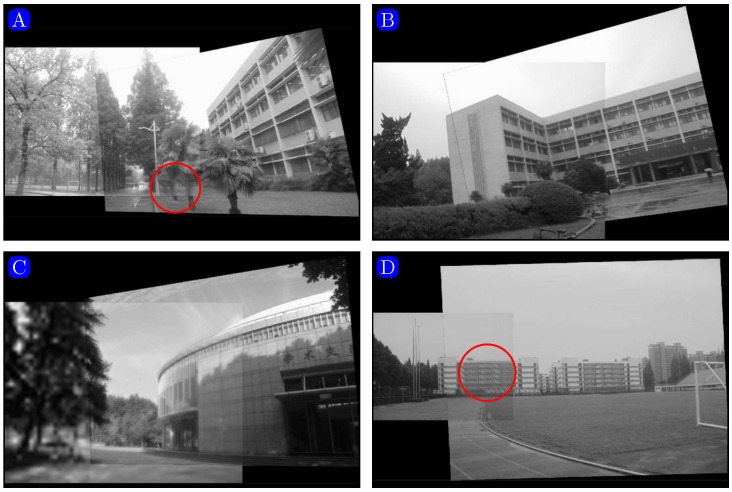
Mosaicking results given by the method proposed in [30]. Reprinted from [30] under a CC BY license, with permission from [Computational and Mathematical Methods in Medicine], original copyright [2014].

**Fig 9:**
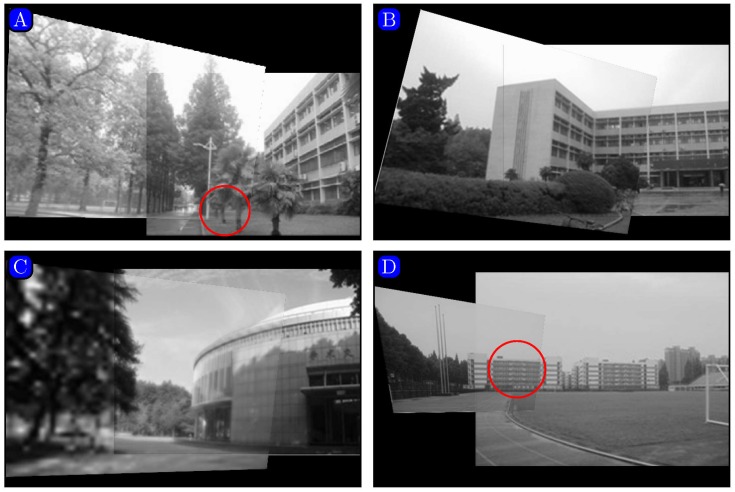
Mosaicking results given by our method.

Figs 8 and 9 show the panoramic images stitched by our method and the algorithm presented in [30]. As shown in Fig 8(A) and 8(D) (in regions marked with red circles), the comparison method results in ghosting due to inaccurate matching.

As shown in Figs 8(C) and 9(C), we can see Fig 9(C) is not clear as the Fig 8(C). The reason is that the quality of the original image downloaded from the website is not good.

**Fig 10:**
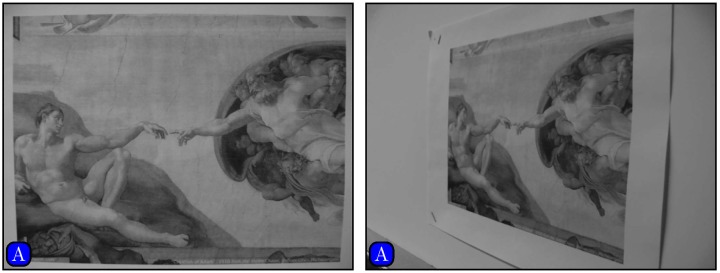
Images with affine distortion.

**Fig 11:**
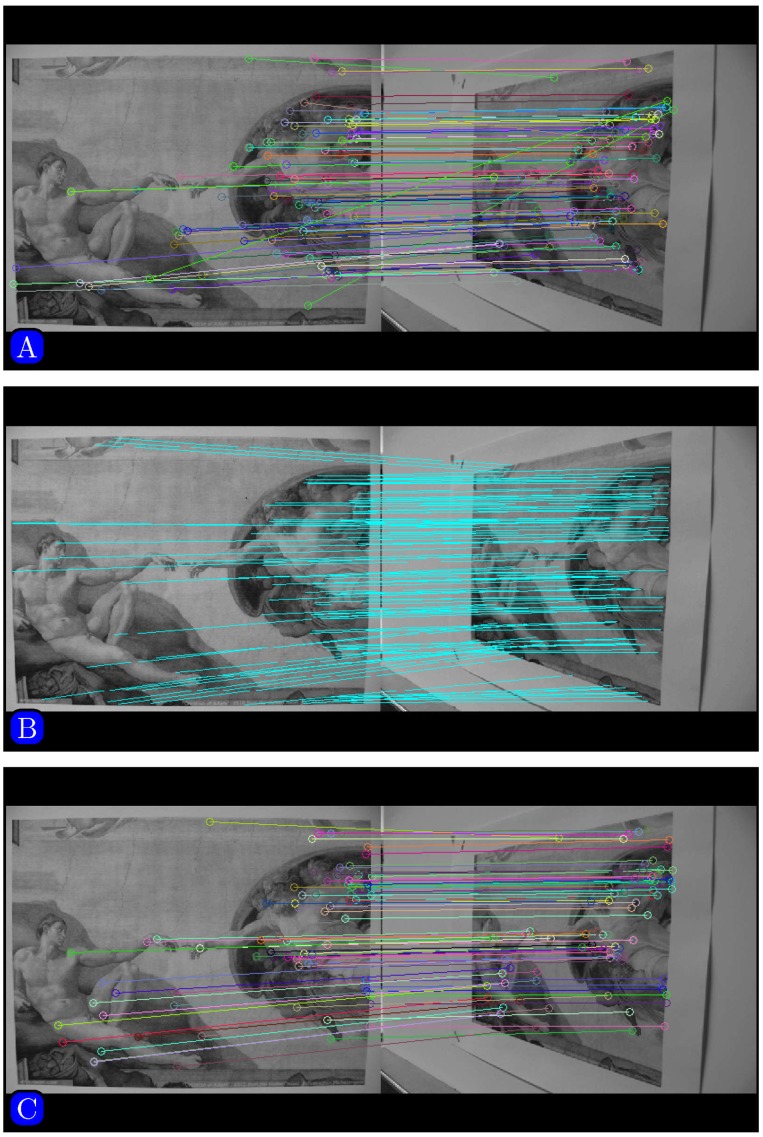
Matching by different methods. (A) SURF, (B) SIFT, (C) Our method.

**Fig 12:**
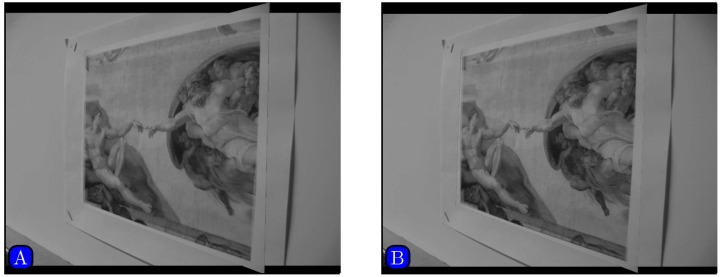
Mosaicking results by different methods. (A) SIFT, (B) Our method.

Fig 10 shows an image pair with significant affine distortion. Results of matching by different methods are shown in Fig 11(A)–11(C). Fig 12 shows the panoramic images stitched by SIFT and our method. We can see that the panoramic image stitched by our method is cleaner than the one given by SIFT.

**Fig 13:**
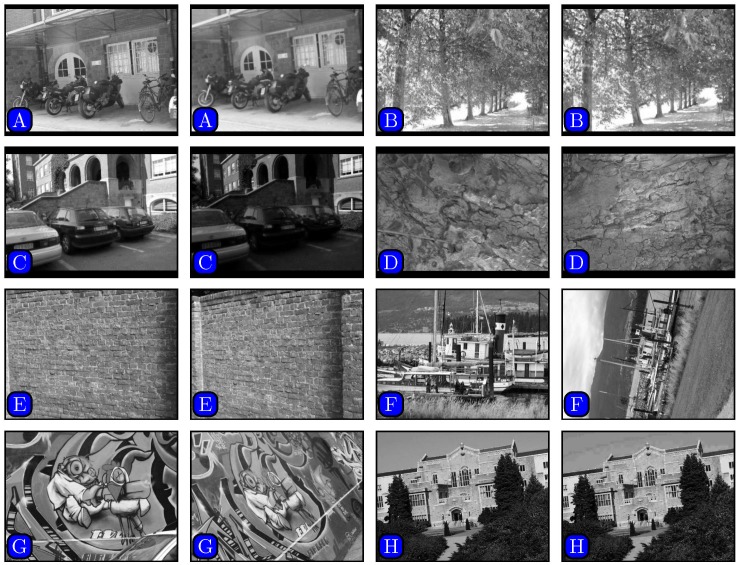
Test image pairs taken from textured and structured scenes under photometric or geometric transformations. (A) Bikes (blur), (B) tree (blur), (C) Leuven (lighting), (D) bark (scaling and rotation), (E) wall brick (viewpoint), (F) boat (rotation), (G) graffiti (viewpoint), (H) UBC (JPEG).

To evaluate the proposed method quantitatively, we used some representative test image pairs from website [36], taken for the textured and structured scenes, as shown in Fig 13. The following metric is used:

$$\begin{array}{c} 1-\text{precision}=\frac{\#\text{false}\;\text{matches}}{\#\text{true}\;\text{matches}+\#\text{false}\;\text{matches}}. \end{array}$$ (8)

Note that a correct match is a match where two keypoints correspond to the same physical location, and a false match is one where two keypoints come from different physical locations.

Table 1 presents the comparison of the matching results, including the number of correct matches over the number of total matches and 1-precision. The results in the table indicate that our proposed algorithm is superior in terms of 1-precision.

**Table 1:** Performance comparison with state-of-the-art methods.

Image	#CM/#TM			1-Precision		
	SURF	SIFT	Proposed	SURF	SIFT	Proposed
A	122/156	234/353	**133/156**	0.22	0.34	**0.15**
B	67/95	140/592	**81/96**	0.29	0.73	**0.16**
C	63/88	97/192	**74/88**	0.28	0.49	**0.16**
D	*	76/156	**28/56**	*	0.51	**0.50**
E	60/133	176/445	**73/133**	0.55	0.60	**0.45**
F	29/62	158/186	**53/62**	0.53	0.15	**0.15**
G	8/23	9/57	**13/23**	0.65	0.84	**0.43**
H	368/422	484/723	**388/422**	0.13	0.33	**0.08**

CM: correct matches; TM: total matches; *: matching failed.

## 5 Conclusion

In this paper, we have introduced a novel image mosaicking method based on SURF features of line segments. This method firstly uses SURF detection operator to detect feature points. Secondly, it constructs directed line segments, describes them with SURF feature, and matches those directed segments to acquire rough point matching. Finally, the RANSAC (RANdom SAmple Consensus) algorithm is used to eliminate incorrect pairs for robust image mosaicking. Experimental results demonstrate that the proposed algorithm is robust to scaling, rotation, lighting, resolution and a substantial range of affine distortion.

Recently, Ji et.al [37] proposed a novel compact bag-of-patterns (CBoP) descriptor with an application to low bit rate mobile landmark search. The CBoP descriptor offers a compact yet discriminative visual representation, which significantly improves search efficiency. In the future, we will try these new methods [37–39] proposed in the fields of mobile visual location recognition and mobile visual search to further improve the performance of our algorithm.

## Supporting information

S1 File Euclidean distances of 10000 matched keypoints. (TXT)
